# Recent Developments in Steelmaking Industry and Potential Alkali Activated Based Steel Waste: A Comprehensive Review

**DOI:** 10.3390/ma15051948

**Published:** 2022-03-06

**Authors:** Ikmal Hakem Aziz, Mohd Mustafa Al Bakri Abdullah, Mohd Arif Anuar Mohd Salleh, Liew Yun Ming, Long Yuan Li, Andrei Victor Sandu, Petrica Vizureanu, Ovidiu Nemes, Shaik Numan Mahdi

**Affiliations:** 1 Faculty of Chemical Engineering Technology, Universiti Malaysia Perlis (UniMAP), Arau 02600, Perlis, Malaysia; arifanuar@unimap.edu.my (M.A.A.M.S.); ymliew@unimap.edu.my (L.Y.M.); 2 Geopolymer & Green Technology, Center of Excellence (CEGeoGTech), Universiti Malaysia Perlis (UniMAP), Arau 02600, Perlis, Malaysia; 3 School of Engineering, University of Plymouth, Plymouth PL4 8AA, UK; long-yuan.li@plymouth.ac.uk; 4 Faculty of Materials Science and Engineering, Gheorghe Asachi Technical University of Iasi, D. Mangeron 41, 700050 Iasi, Romania; peviz@tuiasi.ro; 5 Department of Environmental Engineering and Sustainable Development Entrepreneurship, Faculty of Materials and Environmental Engineering, Technical University of Cluj-Napoca, B-dul Muncii 103-105, 400641 Cluj-Napoca, Romania; 6 School of Civil Engineering, CERSSE-JAIN (Deemed to be University), Bangalore 560069, Karnataka, India; shaik.mahdi@gmail.com

**Keywords:** steel waste, steelmaking, steel waste, alkali-activated cement

## Abstract

The steel industry is responsible for one-third of all global industrial CO_2_ emissions, putting pressure on the industry to shift forward towards more environmentally friendly production methods. The metallurgical industry is under enormous pressure to reduce CO_2_ emissions as a result of growing environmental concerns about global warming. The reduction in CO_2_ emissions is normally fulfilled by recycling steel waste into alkali-activated cement. Numerous types of steel waste have been produced via three main production routes, including blast furnace, electric arc furnace, and basic oxygen furnace. To date, all of the steel waste has been incorporated into alkali activation system to enhance the properties. This review focuses on the current developments over the last ten years in the steelmaking industry. This work also summarizes the utilization of steel waste for improving cement properties through an alkali activation system. Finally, this work presents some future research opportunities with regard to the potential of steel waste to be utilized as an alkali-activated material.

## 1. Introduction

Steel is a globally traded commodity that is manufactured all over the world. It is worth noting that, in 2019, 88% of steel produced in the EU (139 million tonnes) was traded outside of the country of origin, with 111 million tonnes (70% of production) traded on the EU internal market and 28 million tonnes (18% of production) exported outside of the EU, primarily to other European countries (9 million tonnes) and North America (6 million tonnes). Despite producing half of the world’s steel (996 million tonnes), China only export 6% of its output (64 million tonnes), mainly to other Asian countries in 2019 [1]. In comparison, China’s crude steel production reached 627 million tonnes in 2010, demonstrating that steel is in great demand as a result of growing industrialization and urbanization [2].

The steelmaking industry has become the second-largest energy consuming process in global industrial sectors and emits huge amounts of environmentally harmful substances, such as dust, sulphur dioxide (SO_2_), nitrogen oxides (NO_x_), and carbon dioxide (CO_2_). Since the majority of steelmaking operations are still coal-based and heavily reliant on fossils fuels, such as oil and diesel, significant volumes of CO_2_ emissions are emitted. As a result, the steel industry contributes about 6.7 percent of the total global CO_2_ emissions [3]. The manufacturing sector in the EU is responsible for 4.7% of total CO_2_ emission (182 million tonnes) and about 27% of CO_2_ emissions from the worldwide manufacturing sector [4,5]. Steel production emits roughly 1.8 t CO_2_ per tonne, while the total energy demand of steel production is 21.0–35.4 GJ/t steel [6].

**Figure 1:**
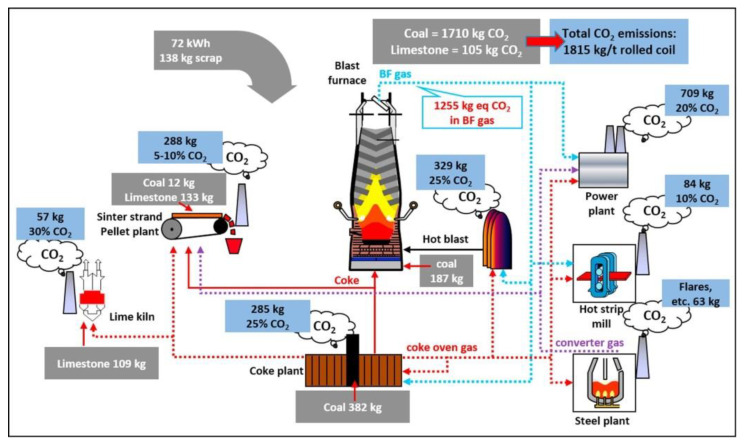
CO_2_ emissions in an integrated steel mill [7].

Steel and iron are manufactured from the metallurgical industry, which is classified into three major routes namely blast furnace (BF), basic oxygen furnace (BOF) and electric arc furnace (EAF). The integrated steel production BF-BOF route is the most crucial steel production route, accounting for roughly 70% of global steel production. The mini-mill approach, which accounts for 25% of global steel production, comprises of EAF in which recycled steel crap is melted and then cast into semi-finished slab, billet or bloom form. As shown in Figure 1 [7], the BF-BOF routes produces one tonne of hot-rolled coil, while emitting approximately 1.8 tonnes of CO_2_. The iron-making processes of blast furnace, sintering, and coke making account for about 90% of the total. The steelmaking pathway has the largest energy consumption and associated CO_2_ emissions, with 12.31 Gj/tHM and 1.22 t CO_2_/tHM [8].

Pollution is produced by all industrial activity, and the steel industry is no exception. Steelmaking and galvanizing processes generate a wide range of waste with varying class, volume, and toxicity. Despite substantial attempts by these industrial sectors to reduce global environmental impacts, there is a constant demand for new technology fresh technology to reduce CO_2_, boost efficiency in recycling waste, and produce clean gaseous and liquid effluents. In fact, environmental control has become a study and technique in the metallurgical engineering industry. According to a study by Pardo and Moya [8], the CO_2_ reduction achievable by 2030, while maintaining the competitiveness of the European steel sector, is in the range of 14–21%, compared to 2010. This necessitates both the incremental development of existing technologies and the incorporation of new, cutting-edge technologies. The future prices of fuels, energy, and other resources, as well as carbon pricing, will have a significant impact on the adoption of these revolutionary technologies. In the economic scenario, the steel sector’s specific, CO_2_ emissions in 2050 would be about 15% lower than in 2010. The European steel industry’s highest specific CO_2_ emission reduction potential in 2050 compared to 2010 is roughly 57%. For this to be realized, all blast furnaces (BFs) would have to be retrofitted with top-gas recycling and carbon capture and storage (CCS) [9].

The solid waste management of steelmaking entails a more complex procedure that aims to limit the quantity of waste that ends up in landfill or is incinerated. It comprises environmentally sound strategies for preventing, reusing, and recycling garbage, as well as the recovery of resources and energy whenever possible. It is preferable to include solid waste in the steelmaking process itself, and then sell it as a raw material for other industrial processes. Instead, the waste could be processed to minimize toxicity and recover commercially valuable materials. Only a small portion of the material is used in this case, with the rest being sent for final disposal as tailing or incineration as required by environmental regulations.

The alkali activation system can be brought by recycling the steel waste to manufacture eco-friendly cement with more exceptional properties than conventional cement. The utilization of steel waste into alkali-activated cement enhances the mechanical and chemical properties. It is noteworthy to highlight that various types of steel waste can be categorized into blast furnace steelmaking waste, electric arc furnace steelmaking waste, and basic oxygen furnace waste (Table 1).

This review focuses on the previous research works on the utilization of varied steel waste in the steelmaking industry. Moreover, cost analysis and energy consumption will be discussed in this paper. In particular, sintering returns approximately 80–90% of mill scale steelmaking processes, while 85–90% of slags are commercialized to other industrial process [28]. Previous papers addressed a wide range of steel manufacturing pathways, from economic and environmental aspects to technological highlights [3,29,30,31]. This review, in contrast, will concentrate on alkali-activated cement application in the most often used integrated steel production pathway. Steel with a high recycling potential necessitates the implementation of long-term management techniques. Meanwhile, there are no recycling options in the cement sector, but cutting energy use and employing alternative fuel sources that produce fewer emissions can assist the industry in becoming more sustainable. The alkali activation method with varied steel waste from the steelmaking sector can be implemented for a reduction in greenhouse gases in the environment. Eventually, additional research opportunities have been offered based on the gaps discovered in the previous literature.

## 2. Steel Production Routes

### 2.1. Steel Waste

Blast furnace or basic oxygen furnace route, melting of scrap in electric arc furnace, direct reduction iron/electric arc furnace, and smelting reduction/basic oxygen furnace are the four primary routes for iron steelmaking. Integrated steel production is the most important steel production route, accounting for roughly 70% of global steel production. The mini-mill approach, which accounts for 25% of global steel production, comprises of EAF in which recycled steel scrap is melted and then cast into semi-finished slab, bloom, or billet forms. The reduction iron/electric arc furnace pathway, which produces around 5% of the world’s steel, primarily uses natural gas as an energy reducing agent. The smelting reduction/basic oxygen furnace approach relies on the burning of coal fines to reduce iron ore fines without agglomeration, and it accounts for only 0.4% of global steel production [32].

The following is a brief overview of the production process in an integrated steel factory, as well as the main forms of solid waste generated at each stage. In order to produce pig iron, the raw material is fed into a blast furnace. Slag forms in the blast furnace, as well as sludge and dust collected in the reactor gas system treatment, are the main type of solid waste generated in this production routes. Meanwhile, the ladle slag and sludge from the gas handling system are two of the common types of solid waste produced in the pig iron refinement process. The refined steel is subsequently delivered to the continuous casting phase for solidification after undergoing a secondary refining step, which take place in the ladle furnace. The steel then acquires the appropriate thickness and mechanical properties during the rolling steel. Additionally, mill scale is a solid waste produced by the oxidation of the metal surface during continuous rolling and casting operations [28]. The numerous residues evaluated in this review, as well as the typical amount produced are listed in Table 2.

### 2.2. Cost Analysis

In the next sections, we evaluate the main technology of blast furnace/basic oxygen furnace regarding process-emission-free and process-emission-intensive technologies in terms of economic costs and process-emission intensities. The statistics are derived from and cross-verified by a variety of sources [29,43,44,45,46], as well as a stakeholder discussion, and refer to a European viewpoint, particularly in terms of resource and energy cost (Table 3).

The most important aspect is that, in terms of operational expenditures (OPEX), direct reduction iron/electric arc furnace steel is around 50% more expensive than blast furnace/basic oxygen furnace steel for the given prices of major elements (labour and capital) and intermediate inputs. Even though usage of direct reduction iron/electric arc furnace reduces costs associated with coke, the iron and steel industry in the analysis provided here converts to hydrogen via water electrolysis using polymer electrolyte membranes. Considering that industrial scale hydrogen generation has yet to be created, unit costs of hydrogen generation vary widely in the literature [47].

Consequently, electricity expenses include both the power required to generate hydrogen and the electricity required for steel production if an electric arc furnace is being utilized [29]. The additional distinction between blast furnace/basic furnace oxygen furnace and direct reduction iron/electric arc furnace is the raw material input, as the latter method requires iron ore to be pre-processed into iron pellets. The remaining cost elements, such as service and primary factor costs, are not significantly different from blast furnace/basic oxygen furnace technology.

## 3. Steel Waste Management

### 3.1. Slags

In a steel mill, the transformation of iron ore into steel produces co-products, or solid wastes, which are classified as slags and sludges. Precipitation sludges, which are generated in the treatment of effluents from galvanizing operations, are among the most prevalent methods for managing such categories of solid waste.

The blast furnace and electric arc furnace are the principal sources of slag in the steelmaking industry. The separation of impurities present in a metal bath, which is constituted of silicates and silicon (Si), aluminium (Al), calcium (Ca), and magnesium oxides (MgO), forms this sort of waste. The chemical make-up of the sources material and the type of refractory employed in the furnace wall determine the concentration of each of these elements [48,49]. After being separated from metal bath, the slag goes through a solidification process. Different kinds of solidification give the material different qualities, resulting in different applications. The two most prevalent procedures are air cooling, which forms a crystalline slag, and granulation, which causes the slag to cool rapidly and become amorphous. Owing to its propensity to absorb water and its feature of hardening after adding water, granulated slag has greater reuse opportunities [28].

Since the volume of slags produced in a steel mill is huge, there are several management options for dealing with this steel waste. In reality, this substance is commonly regarded as a by-product of steel-making production. Table 4 shows a variety of options for managing these wastes.

All of the slags mentioned in Table 4 can be employed in civil construction area, resulting in raw material savings and enhanced mechanical qualities of the finished product. Furthermore, replacing clinker with slag during the manufacturing of cements saves energy since slag does not require a calcination process. This management route also generates an abundance of CO_2_, but using slag may minimize air pollution [28].

Meanwhile, a high expandability of slag related to reactive free oxides elements such as magnesium oxide (MgO) and calcium oxide (CaO) requires an extra care with the use of ladle slag and electric arc furnace slag for such a purpose. This issue can be remedied by ensuring that the material is free of these chemical elements or that any reactions that cause the substance to expand in volume have already occurred [48]. It was pointed out that ladle slag and electric arc furnace slag have a higher iron concentration and typically experience a magnetic separation process to separate the metallic portion (which is recycled in the steelmaking industry) from the non-metallic portion (which is usually allocated to other management routes). Yet, to avoid phosphorus accumulation in the steel, the metallic fraction should have a phosphorus level below than 0.5% [35].

### 3.2. Sludges

Sludges are formed in a wet gas cleaning system, which is used to treat the gases produced during the manufacturing process. Sludges containing zinc (Zn) and lead (Pb) are produced during the manufacturing of pig iron in a blast furnace. Substantial levels of iron (Fe) and carbon (C) are also present in the sludges’ particles, which might be recycled in the furnace [63]. The blast furnace and ladle sludges are the most typical sludges produced in the steelmaking industry. These sludges are mainly composed of calcium, silicon, iron, manganese, and aluminium oxides. The amount of iron in these sludges is usually rather significant [64]. Table 5 shows the various options for dealing with these sludges.

Considering the large proportion of iron oxide in blast furnace and ladle sludges (around 70%), the current trend in managing this type of steel waste is to reuse/recycle it in the steel industry through procedures such as sintering [70]. The inclusion of chemical components such as zinc, on the other hand, hinders direct recycling. Typically introduced as a raw material into blast furnace, zinc elements react with the refractory material, forming a crust around the reactor walls. Furthermore, zinc and lead have been reported to concentrate in the fine portion of the sludge [65]. The zinc-rich sludges from the overflow are stored or landfilled, while the sludge from the underflow is reused in the sinter plant. Conversely, pyrometallurgical or hydrometallurgical processes can be used to eliminate zinc from sludges in a very efficient and cost-effective manner [71].

Hydrometallurgical processes offer greater plant flexibility and correspond to be more cost-effective than pyrometallurgical technologies due to reduced capital and operational cost. By comparing to pyrometallurgy, hydrometallurgical process offer environmental benefits since no off-gases or dust nuisance are identified; nonetheless, effluents developed by these processes should be adequately handled [72].

To summarize, most of steel waste in the last ten years could be recycled/reused in various applications, especially in civil construction field. However, most of the management routes cause a detrimental impact on the environmental and mechanical qualities. Hence, another innovative route has been suggested. For example, steel waste could be utilized as an alkali-activated material in the production of greener construction materials [73,74,75].

### 3.3. Incorporating Alkali-Activated Cement Based Steel Waste

This section primarily focuses on a research project involving the incorporation of steel waste into alkali-activated materials, with the goal of examining the possibility of using an alkali activation system to enhance the mechanical, thermal, and chemical properties of alkali-activated based-steel waste.

One or more alumina-silicate sources and one or more alkaline activators make up an alkali-activated system. The activator solutions promote a pH environment that is in an acidic medium (e.g., silicate, sulphates, carbonated or hydroxides). A pre-mixed alumina-silicate source and alkaline activator in the form of dry powder can be used to produce a dry binder, which can then be mixed with water and aggregates to make mortar or concrete. Alternatively, the alkaline activator solution can be added to the alumina-silicate source separately, and then the wet binder can be mixed with extra water (if the concentration of the alkaline solution needs to be diluted), aggregates and additive materials to build a concrete or mortar. Instead, alkali-activated cement can be produced by mixing alumina-silicate source, alkaline activator, water, aggregates, and admixture without pre-producing the alkali-activated binder [76,77,78].

Any raw materials that consist of major elements of alumina (Al_2_O_3_) and silica (SiO_2_) can be used for alkali activation production. These materials are copiously located in the Earth’s crust and play a crucial role in providing fundamental sources of Al^3+^- and Si^4+^-free cations in the binding system. Commonly, the total compositions of Al_2_O_3_ and SiO_2_ are more than 70% of clay and fly ash material, respectively. Meanwhile, for steel waste, the elements could decrease to the range of 30–50% and mostly appear in the reactive amorphous phase [79]. In this further section, the potential of steel waste minerals in alkali-activated slag cement, mortar, and composites are examined.

### 3.4. Variability of Steel Waste

Most steel waste is often chosen as the aluminosilicate material for alkali activation either as the main or blended binder. The use of steel waste by-products, such as ground granulated blast furnace slag [80,81], steel slag [82,83], nickel slag [84,85] and ladle slag [85,86], may influence these properties due to the variety chemical composition compositions apart from alkali-activated slag cement primary component (SiO_2_ and Al_2_O_3_) as tabulated in Table 6. Steel waste composition varies significantly depending on the type of slag and the stage of steelmaking production. The use of steel waste as an alternative material in alkali activation technology has been extensively studied over previous decades. The studies mainly focused on the effects of different parameters such as Si/Al, Na/Al, SiO_2_/Na_2_O and Al_2_O_3_/Na_2_O molar ratio, slag replacement (weight %), alkali concentration of liquid content, glass content, curing conditions, aggregate size, and slag particle size [87,88,89,90,91].

A unique characteristic of steel waste, the production of alkali-activated materials, has attracted huge attention among researchers. In order to develop better properties of alkali-activated material, steel waste is mixed with other aluminosilicate source materials, such as the binder or filler in the matrix. For example, Samantasinghar et al. [111] incorporated ground granulated blast furnace slag (GGBFS) into class-F type fly ash to enhance compressive strength. A higher availability of leachable alumina-silicates and the presence of calcium oxide (CaO) as one of slag component resulted the strength development as proven by the significant compressive strength. Another study was performed by Gao et al. [112] with the utilization of slag in volcanic ash based alkali-activated. The effect of slag loading (50 and 100%) and activator modulus (0.8, 1.6, and 2.4) in preparing alkali-activated materials on reaction state and chemical environment of molecules were investigated. The higher activator modulus leads to a reduction of slag reactivity. However, it is worth noting that an alkali-activated system with a low CaO concentration can also achieve a high strength (>60 MPa), as stated by Li et al. [113]. A possible explanation for this might be the optimal and correct combination of CaO, Al_2_O_3_ and SiO_2_.

A different study by Bouaissi et al. [114] reported that magnesium plays a role as an addition to calcium in a microstructure, which reflected in the development of mechanical properties. The incorporation of high-magnesium nickel slag (HNMS) in the ground granulated blast furnace slag/fly ash (GGBFS/FA) leads to a strength improvement, resulting in the formation of calcium beryllium praseodymium oxide (CaBePr_2_O_5_), which consists of an orthorhombic crystallography and space group Pnma. The crystalline phase transformation was believed to be attributed to the addition of ground granulated blast furnace slag (GGBFS) and high-magnesium nickel slag (HNMS).

The steel industry generates a vast volume and a diverse range of solid residues, all of which are characterized by a high percentage of metal in structural compositions. Instead of being utilized, these metals burdens are frequently discarded in industrial landfills. As potentially valuable alumina-silicate sources, steel waste from steel industry plants all over the world were characterized using an alkali activation technology. Apart from the use of steel waste in alkali-activated systems, alkali-activated composites, cement and mortar as matrices have also been investigated in the current literature. Since this paper reviews the alkali-activated system for steel waste management, the related works on the use of alkali activator solutions and reaction mechanisms are discussed further.

## 4. Alkaline Activator Solution

Alkali metal solution was used as a liquid component in the alkali activation process. The alkali solution is based on potassium or sodium, which includes hydroxides, alkali silicates, carbonates, and aluminates. The aluminosilicate sources dissolve quickly in a high alkaline environment, releasing AlO_4_ and SiO_4_ tetrahedral components and assisting the polycondensation process [115]. Frequently, the type of alkaline reactant applied in alkali-activated systems is a mixture of hydroxides (KOH or NaOH) and silicates solutions (K_2_SiO_3_ or Na_2_O·nSiO_2_·mH_2_O) [116].

### 4.1. Hydroxide Alkali Solution

Sodium hydroxide (NaOH) and potassium hydroxide (KOH) solutions are widely used for various sources of aluminosilicates due to their leaching ability. Conventionally, a higher concentration of alkali solution accelerates the dissolution of aluminosilicates sources. The dissolution ability of a geopolymer is frequently the determining factor in its ultimate strength. Nevertheless, most geopolymer researchers claimed that a NaOH solution had a better leaching ability than the KOH solution.

Particularly, Sithole et al. [117] tested the unconfined compressive strength (UCS) of slag minerals activated by different activators (KOH and NaOH). As mentioned before, the alkali-activated slag in the NaOH solution possesses higher UCS than the KOH solution. NaOH showed a 45% increment in UCS at similar 15 M concentration compared to KOH. A contradicting result was reported by Altan et al. [118], where they claimed that the KOH activation yields a 10–15% higher compressive strength than NaOH at elevated temperatures. At similar concentrations, KOH contains a higher quantity of solid than the NaOH solution, thus contributing to a higher activating solid-to-slag ratio. Meanwhile, at ambient temperature conditions, the compressive strength of alkali-activated slag in the NaOH solution surpasses the KOH solution. Hence, NaOH activation is preferable over KOH as part of the activator component due to its economic value.

### 4.2. Chemistry of Alkali Hydroxide and Alkali Silicate Solution

As previously mentioned, alkali hydroxide is required at the early stage of alkali activation for the dissolution of aluminosilicates, while alkali silica functions are required as binders or plasticizers [119,120]. Alternatively, silica fumes are often used as supplementary materials to the sodium/potassium silicate. Occasionally, silica fumes are added to enhance the silica species in the design and boost the gelation and silicates precipitation.

Numerous studies on the production of alkali-activated material using only alkali solutions and a mixture of alkali hydroxide and metal silicate liquid were conducted. Most of the comparative studies determined that the chemistry of alkali hydroxide and alkali silicate solution is crucial and developed the preferable microstructure and mechanical properties. The alkali silicate solution induces a unit of soluble SiO_2_ to produce alkali-activated main chain [121]. Based on Singh et al. [122], NaOH-activated slag/blend achieved the optimum compressive strength of 35 MPa at 14 M and had a decrement of up to 16 M. The excess of sodium cation produced sodium carbonate crystal which resulted in an unstable geopolymer edifice.

**Figure 2:**
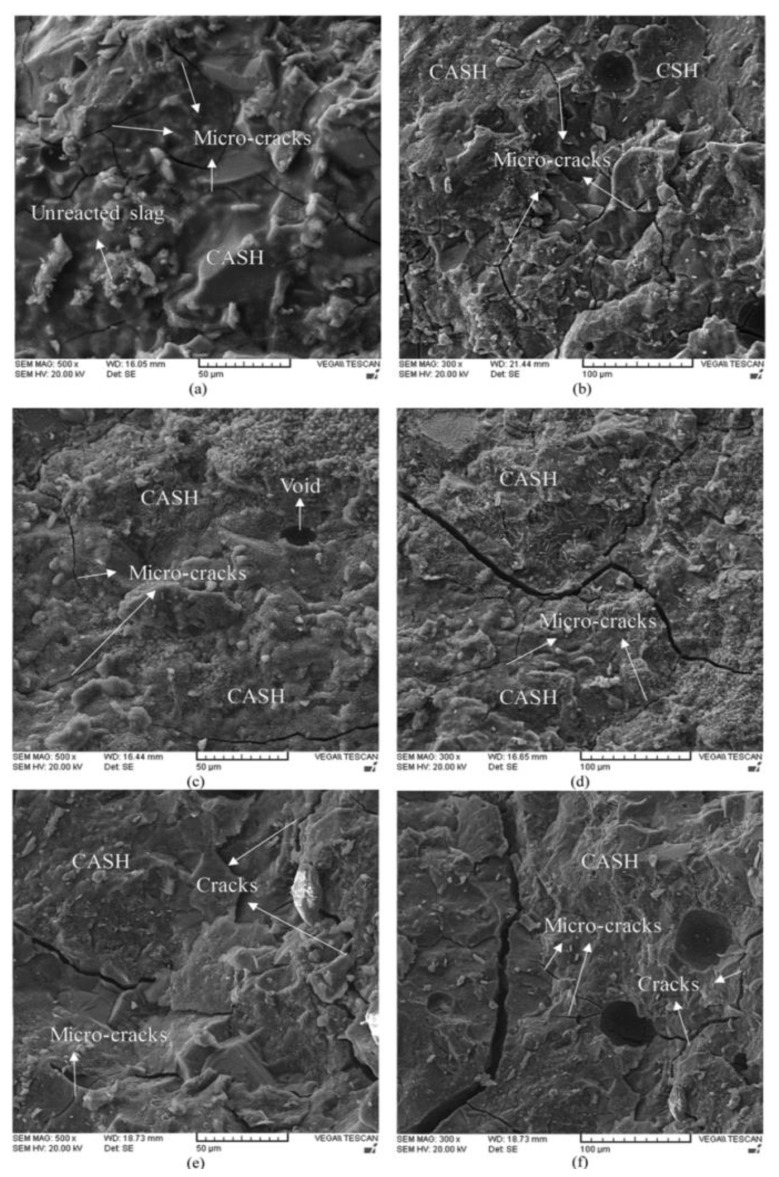
SEM images of AAS paste with various NaOH concentrations at different magnifications: (**a**) 8 M NaOH (500×), (**b**) 8 M NaOH (300×), (**c**) 12 M NaOH (500×), (**d**) 12 M NaOH (300×), (**e**) 16 M NaOH (500×) and (**f**) 16 M NaOH (300×) [123].

Meanwhile, Shariati et al. [123] concluded that the excess of OH^−^ ions during the alkali activation process caused a negative influence, which resulted in higher crack appearance and weak paste structure formation (Figure 2). In alkali-activated systems, increasing NaOH concentration increased the concentration of Na components, which could produce brittle samples once chemically bonded into the main structure of calcium silicate hydrate (C-S-H). Furthermore, according to Cihangir et al. [124], the pore refinements in alkali-activated slag concretes could occur in an acceptable level of Na_2_O concentration. Moreover, increasing the silicate species in alkali activator solutions resulted in a denser microstructure by promoting the chemical reaction between Si and Ca elements in granulated blast furnace slag paste [125]. The denser and more homogenous microstructure is the result of the chemical reaction between silica and calcium components, which suggested the existence of calcium silicate (CaSiO_3_ or Ca_2_SiO_4_).

### 4.3. Reaction Mechanism of Slag Alkali Activation

**Figure 3:**
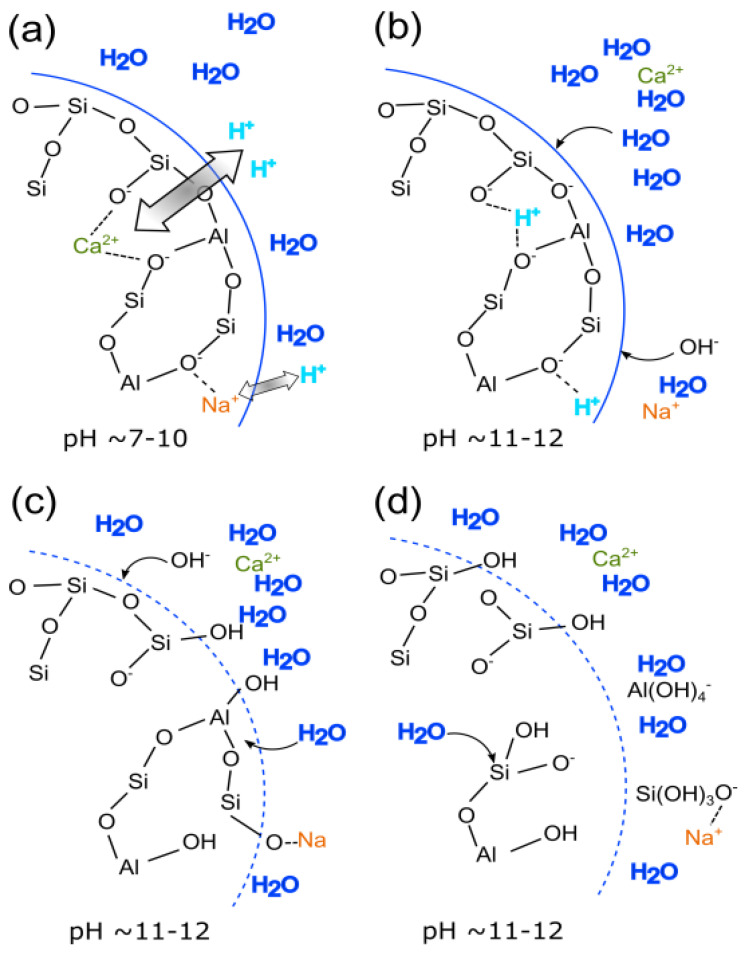
Dissolution mechanism of alkali activation of slag at early stage of (**a**) shifting of H^+^ to Ca^2+^ and Na^+^, (**b**) hydrolysis of Al-O-Si bonds, (**c**) breakdown of the depolymerized glass network, and (**d**) Si and Al detached from the network [126].

Slag has a glassy phase that contains a large amount of calcium, which differs from metakaolin and fly ash in terms of the alkali activation reaction mechanism. Thus, the alkali hydration of a slag corresponds to a complex process that comprises several steps of chemical processes, including the initial dissolution of the slag and polymerization of the final product. As illustrated in Figure 3 [126], the dissolution mechanism of high calcium in the slag system accommodates both divalent and monovalent network-modifying metal cations. The major difference between the Na^+^ and Ca^2+^ illustrates the greater extent of “destruction” caused by the shifting of both monovalent and divalent cations in the main alumina-silicate system.

Slag alkali activation is an exothermic reaction that is similar to other aluminosilicate source materials. The chemical reaction predicted that the process is carried out through either dimers or trimers that allocate the existing component of the 3-D macromolecular structure. The slag alkali activation begins with a destruction of slag bonds Ca-O, Si-O, Al-O, Mg-O, and Fe-O, then produces a stronger Si-Al layer all over the surface of slag grains, ending with hydration products such as tobermorite or calcite.

In the study by Jamil et al. [127] composing oxides components (CaO, SiO_2_, Al_2_O_3_ and MgO) were partly dissolved in the alkaline solution during the early stage of slag alkali activation, indicating that the Ca^2+^ was released from slag and bonded with OH- in alkali solutions to form calcium hydroxide (Ca(OH)_2_), which then reacted with carbon dioxide (CO_2_) in an open environment to form calcite.

**Figure 4:**
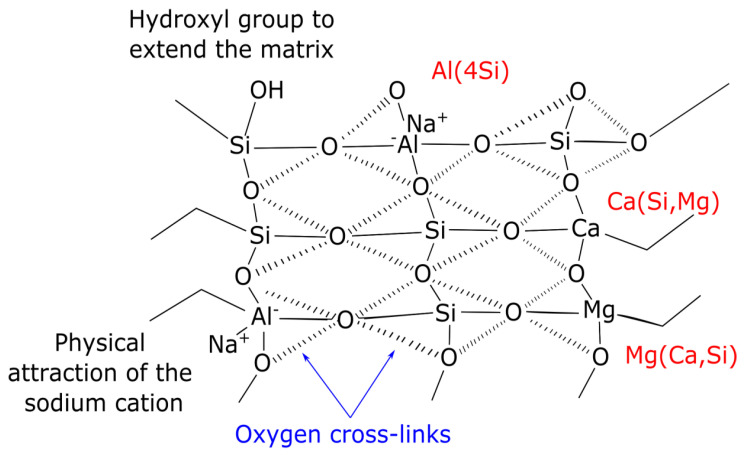
Proposed model of ternary C-A-S-H, and C-M-S-H gel phases [114].

Bouaissi et al. [114] discovered that the free cation Mg^2+^ leads to the formation of intermolecular bonding with Si^4+^ and Al^3+^ by the sharing of oxygen atoms, as depicted in Figure 4. A similar model was proposed by Zhang et al. [128], who also stated that the presence of Mg^2+^ provides chemical stability (interatomic bonding) in the geopolymer matrix as reflected in the formation of Si-O-Mg, Si-O-Al/Si and Ca-O-Si. The reaction mechanism of the alkali activation of slag is more complicated than fundamental geopolymers due to the significant amounts of calcium, magnesium and iron. Hence, it is essential to study the role of these elements in each slag waste material and hydration process.

### 4.4. Alkali-Activated Cement

Alkali-activated cement has been implemented as a replacement material to enhance mechanical properties and fire-resistant abilities, as tabulated in Table 7. Li et al. [129] pioneered the alkali-activated slag to develop high early strength by incorporating a proper mixing of Na_2_CO_3_ and NaOH-Na_2_O·2SiO_2_. The ternary activators not only achieved a reasonable compressive strength, but also obtained the lowest porosity distribution. Another study was performed by Kim et el. [130] with the utilization of the cenosphere in alkali-activated slag cement. The effect of cenosphere replacement (50, 60, and 70%) in alkali-activated slag cement on compressive strength and thermal conductivity were investigated. The use of 70% of the cenosphere was clarified as a floating structural member for freshwater and marine applications.

Aside from its use in enhancing mechanical properties, alkali-activated slag cement has been used to magnify fire-resistance properties. For example, Shahari et al. [134] incorporated fibres into alkali-activated slag cement to enhance their fire-resistance properties. Different types of fibres were incorporated, including polypropylene fibres, basalt fibres, and glass fibres at 0.5%, 1.0% and 1.5%. With the appropriate fibres loading, the compressive strength was developed after been exposed up to 200 °C. Glass and basalt fibre show a better resistance compared to polypropylene fibre owing to superior fire resistance.

### 4.5. Alkali-Activated Mortar

Another reported alkali-activated materials, the implementation of steel waste as alkali-activated mortar (Table 8). For instance, Zhang et al. [135] initiated the utilization of alkali activate mortar with the addition seawater and coral sand. The effect of the modulus of sodium silicate, coral sand/sea sand replacement ratio and water/binder ratio on flexural and compressive strength was investigated. It was pointed out that alkali-activated material produced a hydration product that corresponded to the improvement of the interfacial microstructure between slurry and coral sand. Due to the self-curing of the coral aggregate, the drying shrinkage of the mortar was reduced.

Rovnanik et al. [136] fabricated alkali-activated slag for electrical properties, such as ash resistance and capacitance, and the self-sensing functionality of mortar. The resistivity of alkali-activated slag mortar is nine times lower than that of cement mortar at low AC frequencies; nevertheless, as the AC frequency grows, the resistivity of both materials diminishes, and the values become similar at 500 Hz and above.

Oh et al. [137] found that the superabsorbent polymer is essential for reducing the shrinkage of alkali-activated slag mortar, which is a major limitation in related applications. The role of superabsorbent polymers of storing water inside the matrix initiated the hydration particle reaction.

Therefore, this review classifies the utilization of steel waste as an alkali-activated material with broad applications. The application as a cement replacement, aggregate, mortar and composite material proved that the steel waste could be implemented with any kind of material depending on the desired application.

### 4.6. Alkali-Activated Composites

Aside from alkali-activated cement, another alkali-activated material, such as alkali-activated composites, which consists of fibres, aggregate, and reinforcement materials, is incorporated with steel waste to impart exceptional mechanical properties on composite materials (Table 9). For an example, Nedeljkovic et al. [139] incorporated alkali-activated slag/fly ash and sand aggregates into composites at 2% of polyvinyl alcohol fibres. The contribution of fibres loading resulted in a sufficient bonding between the matrix and fibres. However, fibre failure was obtained for pullout and rupture observations due to fibre rupture. The small amount of fibres limits the capability of mechanical transfer load which believed the fibre rupture at higher strength.

By employing alkali-activated composites, Cui et al. [140] compared the thermal conductivity and mechanical properties of the composites that were incorporated with graphite-modified microencapsulated and carbon fibres. The inclusion of carbon fibres assist in retraining crack pulling and reflected the higher flexural strength of the composites. Meanwhile, the addition of both fibres enhances thermal conductivity.

The most significant importance factor is that the alkali-activated material (inorganic component) could be bonded with an organic component (fibre–polymer type). It is proven that the mechanical and thermal performance of the alkali-activated materials are developed with the acceptable proportion of fibre loadings into composite materials.

## 5. Conclusions and Future Works

In this review, the current development of the steelmaking industry, including cost analysis, energy consumption, and slag variability in the last ten years has been discussed. Additionally, the utilization of steel waste to develop an alkali-activated material has been reported. Their effect on the mechanical and thermal performance of alkali-activated materials is well explained. It was proved that the alkali activation process/technology could be one of the steel waste management in order to solve the landfill problem, environmental issue and economic growth.

Therefore, this review classifies the utilization of steel waste as alkali-activated materials in extensive application. The applications as a cement replacement, aggregate, mortar and composite material proved that the steel waste could be implemented with any kind of material, depending on the desired application. In the steel waste industry, alkali activation can be utilized to create green alternative materials for conventional cement replacement. Hence, the utilization of slag industrial waste should be carried out for the extensive future works, in order to mitigate the disposal nature of steel production and environmental issues. There is an abundance of works involving the manufacturing of alkali-activated slag with exceptional properties for extensive applications. Nevertheless, in order to produce exceptional properties of alkali-activated slag, the details of process chemistry, reaction mechanism, and material characteristic are elaborated. Alkali concentration and liquid/solid ratios are evaluated as the influencing parameters that affect the properties of alkali-activated slag.

Based on the identified gaps in this manuscript, future works on the alkali activation of varied steel waste material are listed below:

- Material and energy flow mechanisms in the steelmaking industry are still poorly understood, especially in the variable setting of steel production. Consequently, the quality of material and energy fluxes, as well as steel waste characteristics, necessitates greater consideration.
- The reaction mechanism and reaction products of alkali-activated cement are contributed to by prime materials and alkaline activators, hence the details of alkali-activation-based steel waste require more attention.
- It is also recommended that the number of steel waste management routes are increased, allowing the environmental impact to be reduced due to the introduction of more efficient technologies. As a result, organizations who embrace such approaches may save costs, add value to industrial waste, and develop the profitability and competitiveness of the manufacturing process.
- The evaluation of landfill cost avoidance benefits as part of production costs is important for the consideration of the impact on the steelmaking industry.

## Figures and Tables

**Table 1:** Type of steel waste from different types of steelmaking production.

Steelmaking Production	Type of Steel Waste	Description
Blast furnace	Ground granulated blast furnace slag (GGBFS)	Cement replacement [10], high-performance concrete [11], electromagnetic performance [12], and steel reinforcement material [13]
	Ladle slag	Supplementary material [14], High-strength cement [15], cement replacement [16], soft clay stabilization [17]
Electric arc furnace	Electric arc furnace slag	One part hybrid cement [18], cement mortar [19], concrete pavement [20], self-compacting concrete [21]
	Steel slag	Alkali-activated cement [22], high-strength cement [23], cement-based composite binders [24]
Basic oxygen furnace	Basic oxygen furnace slag	Cement replacement [25], bacterial community succession [26], cement mortar [27]

**Table 2:** The average of steel waste generated from steelmaking production routes.

Type of Steel Waste	The Average Amount Generated
Blast furnace slag	150 up to 300 kg per tonne of pig iron (blast furnace powered by charcoal) and 200 up to 400 kg per tonne of pig iron (blast furnace fuelled by mineral coal) [33,34]
Ladle slag	Each tonne of liquid steel weighs around 200 kg [35]
Electric arc slag	Approximately 130 up to 180 kg per tonne of [36]
Blast furnace sludge	Precisely 6 kg per tonne of pig iron [37,38]
Ladle sludge	15 up to 16 kg per tonne of hot metals [39,40]
Electric arc dust	15 up to 20 kg per tonne of steel [41]
Mill scale	34 up to 40 kg per tonne of steel [28,42]

**Table 3:** Cost analysis of different iron and steel production routes (net of taxes).

Technology (EUR/t Steel)	Blast Furnace/Basic Oxygen Furnace	Direct Reduction Iron/Electric Arc Furnace
Electricity	0	219
Iron pellets	0	84
Coke	84	0
Iron ore	189	189
Services	45	40
Skilled labour	44	40
Unskilled labour	5	4
OPEX (EUR/t steel)	415	624
Process emission (t CO_2_/t steel)	1.5	-
Investment cost (EUR/t steel)	-	1113

**Table 4:** Management options for steelmaking slags.

Type of Steel Waste	Blast Furnace Slag	Electric Arc Furnace Slag	Ladle Slag	References
Management Options				
Reuse/recycling in steelmaking	-	Roughly 30% of slag is recycled in blast furnace in European countries; however, the phosphorus concentration should not exceed 0.5%. The elimination of phosphorus element is still a subject of research	-	[35,50]
Utilize as aggregates	The samples were maintained in sealed bag for 28 days in a curing environment at a temperature of 21 °C and relative humidity of 70%, providing superior mechanical properties to aggregate slag concrete.	Required the curing process (demoulded after 24 h, then cured at 20 °C of water tank) for because to the high expandability of the electric arc and ladle slag. It is not only cost effective, but it also has advantages in terms of material properties		[51,52,53]
Conventional cement manufacture	Owing to the hydraulicity of granulated slag, the residue used as a partial replacement for clinker material that leads to lower raw material and energy consumption, reduced pollution in cement manufacturing and enhanced finished material qualities. All the samples were cured in the range temperature of 20–35 °C	These residues obtain lesser hydraulic characteristics than blast furnace slag and can replace a portion of the clinker. Additionally, due to the expandability properties, such slags should go through the curing procedure for 28 days		[49,54,55]
Catalyst for the manufacture of biofuels	The effective catalyst for the synthesis of biodiesel was proven due to the slag crystallinity			[56,57,58]
Manufacturing of glass ceramic	The utilization of steel waste is widely known and commonly used. Glass ceramic structures are formed by the crystallization vitreous materials, such as slag under regulated conditions.			[59,60]
Absorbent materials	Higher reactivity and better specific surface area was obtained by slag materials when compared to the conventional absorbent			[61,62]

**Table 5:** Management options for steelmaking sludges.

Type of Steel Waste	Blast Furnace Sludge	Ladle Sludge	References
Management Options			
Reuse/recycling in steelmaking	Lead and zinc must be eliminated from the dry sludges before they may be recycled directly. Since the majority of these elements are concentrated in the fine fraction, the coarser fraction of sludges could be recycled after classification during the steelmaking process. The reuse of the fine fraction still necessitates further research into removal of undesirable materials.		[37,39,65]
Utilize as adsorbent material	Preferably an effective adsorbents for copper, zinc, lead, chromium, and cadmium in various concentrations	-	[66,67]
Ceramic materials incorporation	As a result of the process, energy is saved, and waste disposal cost is reduced.		[68,69]

**Table 6:** Chemical composition of slag from different region and sources.

Type of Slag	Chemical Composition (wt %)				
	SiO_2_	Al_2_O_3_	CaO	MgO	Fe_2_O_3_
Steel slag (Shandong Sheng, China) [92]	19.13	4.87	37.42	5.55	18.77
Steel slag (Wuhan, China) [89]	15.0	6.7	44.2	10.9	15.4
Steel slag (Wuhan, China) [83]	15.1	2.32	42.98	5.77	21.13
Steel slag (Jiangxi, China) [93]	18.48	3.76	45.18	4.83	19.45
High-magnesium slag (Nanjing, China) [84]	52.3	6.2	8.8	26.9	4.2
High-magnesium nickel slag (Jiangsu, China) [93]	50.37	4.65	1.72	32.22	7.94
Copper nickel slag (Murmansk, Russia) [94]	36.87	7.44	2.11	11.92	2.47
Copper nickel slag (Xinjiang, China) [95]	29.68	1.473	3.253	6.212	55.45
Copper slag (Aspropyrgos, Greece) [96]	39.95	3.30	4.08	1.77	44.41
Ferronickel slag (Larymna, Greece) [97]	32.74	8.32	3.73	2.76	0.76
Ferronickel slag (Marousi, Greece) [98]	40.29	10.11	3.65	5.43	37.69
Ferronickel slag (New Caledonia. France) [99]	52.52	2.33	0.27	33.16	10.80
Ferrochrome slag (Elazig, Turkey) [100]	33.8	25.48	1.1	35.88	-
Ferrochrome slag (Bhubaneswar, India) [101]	27.8	23.6	3.51	23.7	3.6
Ferrochrome slag (Malatya, Turkey) [102]	33.80	25.48	1.10	35.88	0.61
Ground granulated blast furnace slag (Chhattisgarh, India) [103]	32.97	17.97	35.08	10.31	0.72
Granulated blast furnace slag (Melbourne, Australia) [104]	33.8	13.68	42.56	5.34	0.4
Ground granulated blast furnace slag (Paris, France) [105]	35.7	11.21	39.4	10.74	0.42
Granulated blast furnace slag (Dabrowa Goronicza, Poland) [106]	38.73	8.18	45.09	4.33	0.90
Granulated blast furnace slag (Cairo-Egypt) [107]	36.95	10.01	33.07	6.43	1.48
Blast furnace slag (Jiangsu, China) [108]	34.2	14.2	41.7	6.7	0.43
Ladle furnace slag (Taipei, Taiwan) [86]	23.7	4.2	48.6	8.1	-
Ladle slag (Lappohja, Finland) [109]	8.6	28.3	46.3	7.4	5.0
Blast oxygen furnace (Indiana, USA) [110]	8.35	60.8	5.21	8.89	2.35

**Table 7:** Research work utilizing alkali-activated slag cement.

No	Researcher	Materials	Findings
1	Kim et al. [130]	Blast furnace slag and cenosphere	Increase water absorption rate Decrease density, compressive strength and thermal conductivity
2	Li et al. [129]	Ground blast furnace slag and river sand	Shortened initial setting time Increase compressive strength
3	He at al. [131]	Ground blast furnace slag, water glass and hydrated lime	Increase compressive strength at early age Decreased drying shrinkage
4	Hyeok-Jung et al. [132]	Ground granulated blast furnace slag and red mud	Increase compressive strength Increase efflorescence area
5	Nikolic et al. [133]	Electric arc furnace slag and electric arc furnace dust	Deterioration of mechanical properties Higher porous structure

**Table 8:** Research work utilizing alkali-activated slag mortar.

No	Researcher	Materials	Findings
1	Zhang et al. [135]	Ground granulated blast furnace slag, fly ash, silica fume, coral sand	Reduction in drying shrinkage Higher mechanical performance
2	Rovnanik et al. [136]	Slag, quartz, cement	Large number of micropores Remarkable self-sensing properties
3	Oh et al. [137]	Portland cement, superabsorbent polymers, and granulated blast furnace slag	Significant reduction in shrinkage Higher compressive strength
4	Kumarappa et al. [138]	Slag cement and shale lightweight aggregate	The development of autogenous shrinkage can be controlled Reduce surface tension

**Table 9:** Research work utilizing on alkali-activated slag composite.

No	Researcher	Materials	Findings
1	Nedeljkovic et al. [139]	Slag/fly ash, sand aggregates, polyvinyl alcohol fibers	Stronger bond formed between matrix and fibres Limit the capability of mechanical transfer load
2	Cui et al. [140]	Ground granulated blast furnace slag, polycarboxylate, graphite-modified microencapsulated, and carbon fibre	Higher compressive strength compared to conventional cement Lower thermal conductivity Achieve good thermal storage
3	Jiape et al. [141]	Ground granulated blast furnace slag, cement and epoxy resin	Longer setting time required Good bond and uncracking microstructure
4	Kan et al. [142]	Incineration fly ash, ground granulated blast furnace slag, polycarboxylate-based high-range water reducing mixture	Better mechanical properties Larger tensile strain capacity Good for immobilizing toxic heavy metals
5	Cristelo et al. [143]	Steel slag, fly ash and silica sand	Obtain superior mechanical performance Well-graduated transition zone developed

## Data Availability

Not applicable.
